# Experimental and Numerical Investigation of the Micro-Crack Damage in Elastic Solids by Two-Way Collinear Mixing Method

**DOI:** 10.3390/s21062061

**Published:** 2021-03-15

**Authors:** Hongjun Liu, Youxuan Zhao, Han Zhang, Mingxi Deng, Ning Hu, Xiaoyang Bi

**Affiliations:** 1 College of Aerospace Engineering, Chongqing University, Chongqing 400044, China; liuhongjun@cqu.edu.cn (H.L.); mxdeng@cqu.edu.cn (M.D.); 2 Chongqing Key Laboratory of Heterogeneous Material Mechanics, Chongqing University, Chongqing 400044, China; 3 Key Laboratory of Noise and Vibration, Institute of Acoustics, Chinese Academy of Sciences, Beijing 100190, China; zhanghan@mail.ioa.ac.cn; 4 State Key Laboratory of Reliability and Intelligence Electrical Equipment, Hebei University of Technology, Tianjin 300401, China; 5 National Engineering Research Center for Technological Innovation Method and Tool, Hebei University of Technology, Tianjin 300401, China; 6 School of Mechanical Engineering, Hebei University of Technology, Tianjin 300401, China; xy_bi1001@126.com

**Keywords:** ultrasonic nonlinearity, wave mixing, micro-cracks, experiment, numerical simulation

## Abstract

This study experimentally and numerically investigated the nonlinear behavior of the resonant bulk waves generated by the two-way collinear mixing method in 5052 aluminum alloy with micro-crack damage. When the primary longitudinal and transverse waves mixed in the micro-crack damage region, numerical and experimental results both verified the generation of resonant waves if the resonant condition $\omega_{L}/\omega_{T}=2\kappa /\left(\kappa -1\right)$ was satisfied. Meanwhile, we found that the acoustic nonlinearity parameter (ANP) increases monotonously with increases in micro-crack density, the size of the micro-crack region, the frequency of resonant waves and friction coefficient of micro-crack surfaces. Furthermore, the micro-crack damage in a specimen generated by low-temperature fatigue experiment was employed. It was found that the micro-crack damage region can be located by scanning the specimen based on the two-way collinear mixing method.

## 1. Introduction

Due to the influence of fatigue loading, micro-cracks can be easily initiated in metallic material, which extends and degrades the material’s performance. The development and aggregation of micro-cracks can generate a macro-crack, which leads to the final fatigue failure. Thus, it is of great importance to detect and evaluate micro-crack damage in materials at an early stage to ensure the safety of engineering structures.

As one of the most important nondestructive testing methods, ultrasonic testing is widely used in various industries because it is nondestructive, highly efficient and cost-effective. In particular, nonlinear ultrasonic techniques [1,2] have drawn much attention in recent years for their high sensitivity to material microstructural changes. Meanwhile, the feasibility of nonlinear ultrasonic techniques for detecting and evaluating early material degradation has been widely demonstrated by theory, simulation and experiment. As representative nonlinear ultrasonic techniques, higher harmonics technology [3,4,5,6,7,8,9] and wave mixing technology [10,11,12] have been commonly developed. The average or equivalent material nonlinearity in wave propagation paths can be tackled by higher harmonics technology. For instance, Shui et al. [13] experimentally applied second harmonics of longitudinal wave to evaluate impact fatigue damage in adhesive bonding. Herrmann et al. [14] proposed a reliable technique based on nonlinear Rayleigh surface waves to assess material damage at different stages of fatigue life. Lim et al. [15] numerically and experimentally studied the generation of second harmonics caused by the interaction between Lamb waves and a fatigue crack. Compared with higher harmonics technology, wave mixing technology has some advantages, such as its feasibility for scanning local damage, and its flexible frequency selection to avoid nonlinear interference from the electronic system. Chen [16], Gao [17] and Liu et al. [18] derived the necessary and sufficient resonant conditions of two propagating time-harmonic plane waves with the various resonant types with material nonlinearity. Zhao et al. numerically and experimentally investigated one-way bulk wave mixing behavior based on material quadratic nonlinearity [19], and they numerically investigated the detection of the micro-crack damage using the one-way collinear mixing method [20]. Tang [12,21] and Shui et al. [22] employed the two-way collinear mixing method to evaluate and locate plastic damage. Jiao et al. [23] employed a collinear wave mixing of two longitudinal waves to detect a single micro-crack. Jacobs et al. [24] analytically investigated the nonlinear mixing mechanism of two collinear Rayleigh waves in isotropic nonlinear elastic solids. Sun et al. [25] derived the resonant condition for one-way mixing of nonlinear Lamb waves to identify mode triplets. Based on the condition of phase velocity matching, Lissenden et al. [26,27,28] investigated the generation and propagation mechanism of Lamb mixing behavior with multiple modes. Li et al. [29] studied the acoustic nonlinear behavior from third-order Lamb-mixing harmonics. Hu et al. [30,31] investigated the one-way collinear mixing of *A*_0_ and *S*_0_ mode Lamb waves with quadratic nonlinearity and randomly distributed micro-cracks. In addition, the non-collinear ultrasonic wave mixing technique [32,33,34,35,36,37] has also been studied by some researchers. Xiang [36], Croxford [38], Mao [39] and Jiao et al. [40,41] employed non-collinear two shear waves mixing method to detect plasticity, fatigue and a single fatigue crack. Ishii et al. [34,42] analytically and numerically investigated the modal amplitude in the non-collinear interaction of guided waves. Wang et al. [43,44] proposed analytical models to predict nonlinear mixing of non-collinear guided waves and bulk waves at a contact interface, respectively.

Most of previous theoretical studies have focused on the evaluation of the material degradation based on quadratic nonlinearity. In fact, the interaction between ultrasonic waves and micro-cracks can lead to the nonlinear effect [45,46,47]. Three constitutive models of micro-cracks are mainly considered, including the hysteresis model [48], bi-linear stiffness model [49,50] and the rough surface contact model [51,52]. Moreover, research has been devoted to the evaluation of micro-cracks in recent years. Qu et al. [53] analytically explained the phenomenon of nonlinear interaction between bulk waves and micro-cracks. Sun et al. [54] derived the acoustic nonlinearity parameter (ANP) based on the zero-frequency component and second harmonics. Hu et al. [55] systematically studied the generation mechanism of nonlinear Lamb waves in the micro-crack region based on the low-frequency *S*_0_ mode Lamb wave method. Su et al. [56] illuminated the generation mechanism of contact nonlinearity based on a three-dimensional crack “breathing” model. Jiao et al. [57] employed a nonlinear Lamb wave mixing technique to evaluate the length and width of the crack in a plate.

Due to the difficulty of manufacturing coaxial double crystal transducers, the realistic application of the one-way collinear mixing method has been severely restricted. In contrast, the two-way collinear mixing method only needs two independent transducers, and the applicability and flexibility of this method can be assured. However, research on micro-crack damage and the formulas for locating the damage region (plastic or micro-crack) based on the two-way collinear mixing method is seldom found in the literature. For instance, in [16,17,18], the resonant condition of the two-way collinear mixing method is only considered for material nonlinearity, but not the micro-crack damage; in [12,21,22], the plastic damage, instead of the micro-crack damage, is evaluated and located using the two-way collinear mixing method. Thus, different to previous studies on bulk wave mixing methods, this paper focused on the detection and evaluation of the micro-crack damage based on the two-way collinear mixing method using numerical simulation and experimental measurement. The aim of this work was to numerically investigate the variation characteristics between resonant waves and the micro-crack damage based on simplified numerical modelling, and to experimentally verify the feasibility of this two-way collinear mixing method for the detection of micro-crack damage in a specimen. The quantitative relationships between the ANP and the key factors of micro-crack characterization were numerically studied, and the expressions to calculate the position of the micro-crack damage region were proposed. In addition, locating the micro-crack damage was also conducted by experimental measurement, which was observed by surface electron microscopy (SEM) after a low-temperature fatigue experiment. In summary, the novelties of this work could be: (1) the resonant condition of the two-way collinear mixing method for the micro-crack damage was first investigated by numerical simulations, which were correspondingly verified by ultrasonic scanning experiment; and (2) formulas for locating the micro-crack damage region in the two-way collinear mixing method were proposed and verified.

## 2. Numerical Simulations

### 2.1. Resonant Condition

When a transverse wave pulse is emitted at x=0 and propagates in the positive *x*-direction while a longitudinal wave pulse is emitted at x=L and propagates in the negative *x*-direction, it is called two-way mixing. If the resonant condition $\omega_{L}/\omega_{T}=2\kappa /\left(\kappa -1\right)$ [16] is satisfied and there is material nonlinearity, a resonant transverse wave with the difference frequency of the two primary waves can be generated, which propagates in the negative *x*-direction, where $\omega_{L}$ and $\omega_{T}$ are the circular frequencies of longitudinal and transverse waves, respectively; $\kappa =c_{L}/c_{T}$, with $c_{L}$ and $c_{T}$ being the longitudinal and transverse phase velocities, respectively.

It should be noted that the above resonant condition is derived for the quadratic nonlinearity, and we suppose that the resonant condition is available for the micro-crack damage model [20]. Numerical simulations were then employed to validate the feasibility of the above assumption.

### 2.2. Numerical Modelling

In order to investigate the generation mechanism of the resonant wave and the relationship between the ANP and the micro-crack damage, the simplified two-dimensional plane strain model with periodic boundary conditions was employed to describe wave propagation in elastic solids with micro-cracks. Note that we only considered the existence of micro-cracks rather than the generation and evolution of micro-cracks. The representative volume element (RVE) method was also adopted to establish the region of the micro-crack damage. Meanwhile, the contact behavior was modelled as the source of the nonlinearity from micro-cracks in finite element models (FEM), and the material property was regarded as ideal linear elasticity. The commercial FEM software ABAQUS (Version 6.14, Dassault Systèmes Simulia Corp., Providence, RI, USA) was adopted to establish the FEM models with randomly distributed micro-cracks. A similar micro-crack modelling method presented by Zhao et al. [20,30,54] was employed to simulate the two-way collinear wave mixing in the micro-crack damage region.

**Figure 1:**
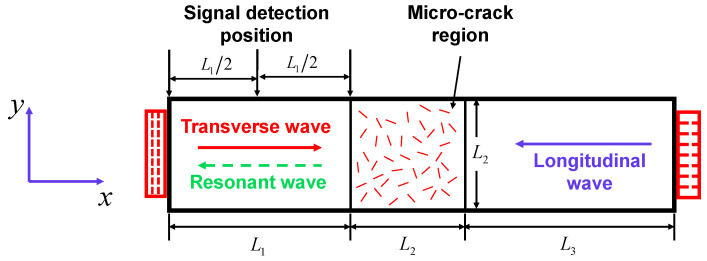
Schematic of two-way collinear mixing in an elastic solid with randomly distributed micro-cracks.

By successively emitting a pair of transverse and longitudinal waves at two opposite sides, a model of two-way collinear bulk wave mixing in an elastic solid with randomly distributed micro-cracks was created, and is described in Figure 1. The micro-crack damage region with the size of *L*_2_ × *L*_2_ is in the middle of the model. The distance between the left boundary of the damage region and the left edge of the model is *L*_1_, and the distance between the right boundary of the damage region and the right edge of the model is *L*_3_. Besides, three signal detection positions are equally located with the same interval *L*_1_/2 in the left of the model. To simulate the emission of transverse and longitudinal wave pulses, two different dynamic displacement excitations were employed on the left and right edges of the model, respectively. Due to the different velocities between the transverse and longitudinal waves, the longitudinal wave pulse should be generated after the transverse wave pulse. The two primary pulses propagate in the opposite direction of *x*, arrive and interact in the micro-crack region. Consequently, if the resonant condition $\omega_{L}/\omega_{T}=2\kappa /\left(\kappa -1\right)$ is satisfied, a resonant transverse wave pulse propagating in the negative direction of *x* can be generated in the micro-crack region and finally received at signal detection positions. Moreover, periodic boundary conditions were enforced on the top and bottom edges of the model to ensure two primary pulses in the form of plane waves.

To investigate the influence of micro-cracks on the generation of resonant waves, *N* micro-cracks with a uniform length of 2*a* (the range of *a* is from 10 μm to 50 μm) were randomly distributed in the micro-crack region. In order to keep the randomness of micro-cracks distribution, the probability density function with a uniformly random variable in numerical simulations was employed to determine the center position and the orientation of cracks. Moreover, the expression $c=Na^{2}/A$ [53] was used for the definition of the crack density, where *A* is the area of the micro-crack region. It should be noted that *a* is much smaller than *L*_2_, indicating that the size of the micro-crack is much smaller than the size of the micro-crack region. Additionally, *L*_2_/*a* = 100 is appropriate for guaranteeing computational accuracy and efficiency [53].

**Figure 2:**
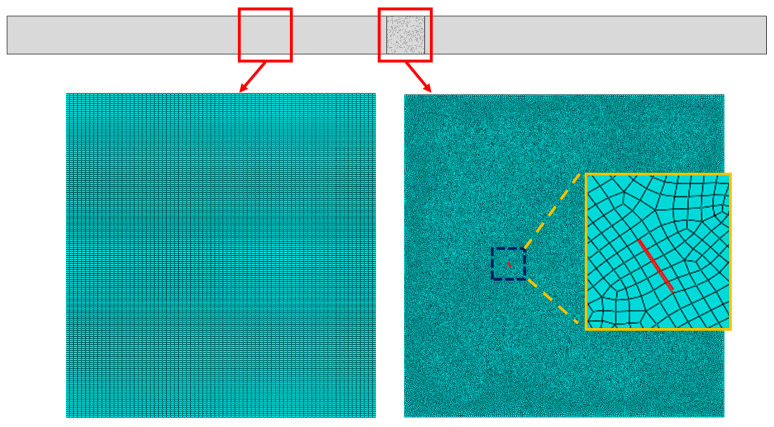
Finite element model (FEM) of the micro-crack region: the distribution of micro-cracks and finite element mesh (the red line represents one crack in the micro-crack region, *L*_1_ = 40 mm, *L*_2_ = 4 mm, *L*_3_ = 36 mm).

The linear elastic constitutive model of the aluminum alloy (Al-5052) was adopted, and the material properties were *ρ* = 2700 kg/m^3^, *E* = 70.0 GPa and *v* = 0.33. To describe the clapping and sliding behavior between micro-crack surfaces, the contact model with “hard contact” in the normal direction and the Coulomb law of friction (the friction coefficient *μ*) in the tangential direction was employed. Next, based on the recommendation [53] for more than 20 linear elements within the shortest wavelength, the element size in the FEM model was set to 0.025 mm, and 5 elements were required in every micro-crack to accurately ensure the interaction between the two primary waves and micro-cracks. Thus, the two-dimensional FEM model measuring 80 × 4 mm^2^ was representatively constructed by 510,000 four-node plane strain elements (CPE4R), as shown in Figure 2.

The transverse and longitudinal waves were generated on the left and right edges, respectively, of the FEM model with the dynamic displacement excitation $u(x,t)=A_{0}\sin(2\pi ft)$, where *A*_0_ is the amplitude of two primary wave pulses. The amplitudes of the longitudinal and transverse waves were 10^−5^ mm and 10^−4^ mm, respectively. The excitation cycle was 10 in the two primary wave pulses. Considering the resonant condition and the assumption of low frequency with respect to the crack size, *f_L_* = 8 MHz and *f_T_* = 2 MHz were adopted for longitudinal and transverse waves, respectively, which leads to the frequency of the resonant wave *f_R_* = *f_L_* − *f_T_* = 6 MHz. Thus, λ/a≈20 in this work results in the assumption of low frequency. Moreover, ABAQUS/Explicit solver based on the central difference method was employed to analyze the generation of the resonant wave. The stable time increment was set to be $\Delta t=5.0\times 10^{-10}$ seconds, considering the need for numerical convergence, accuracy and efficiency.

In addition, the ANP with the expression *β = A_R_*/(*A_L_A_T_*) [57] was adopted to represent the degree of damage of micro-cracks in this study, where *A_R_*, *A_L_* and *A_T_* are the amplitudes of the resonant, the longitudinal and the transverse waves, respectively.

**Figure 3:**
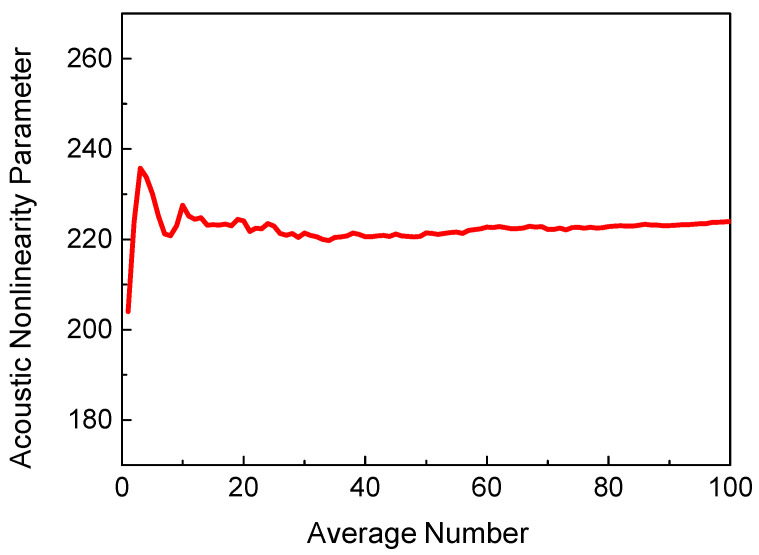
Acoustic nonlinearity parameter (ANP) versus average number of FEM models (*c* = 0.009375, *f_R_* = 6 MHz, *μ* = 0, *L*_1_ = 40 mm, *L*_2_ = 4 mm, *L*_3_ = 36 mm).

Finally, the influence of the random distribution of micro-cracks needs to be considered. To investigate the relationship between micro-cracks’ randomness and the ANP, sufficient FEM results should be averaged. The trend in the averaged ANP with the number of FEM models is shown in Figure 3. When the average number is more than 30, a stable averaged ANP can be achieved. Hence, all results were averaged over 30 FEM models in the following discussion.

### 2.3. Numerical Results

In this section, we investigated the generation and propagation of the resonant wave under the resonant condition and quantitatively reveal the relationship between the ANP and the micro-crack damage. The feasibility of locating the micro-crack damage region was also verified.

**Figure 4:**
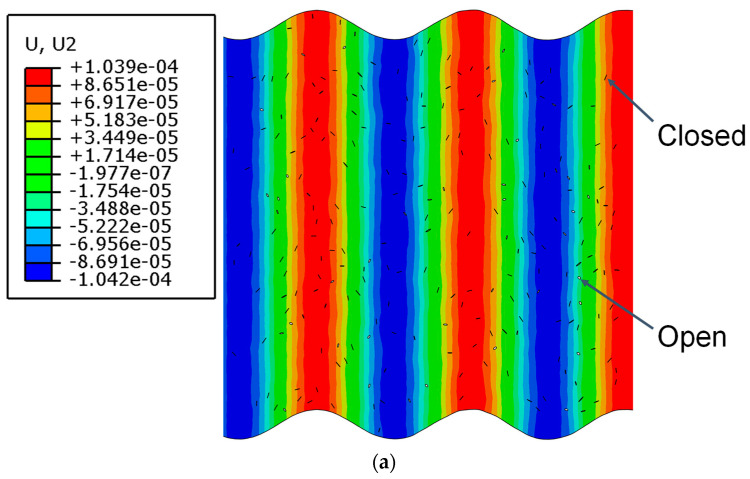

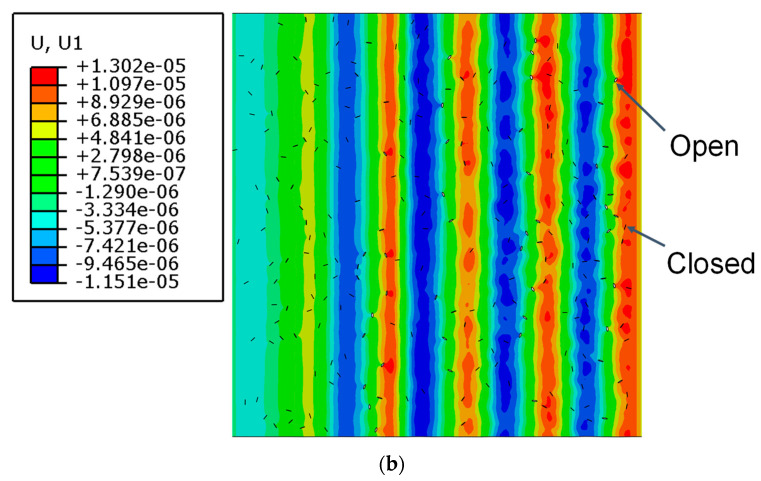
Propagation of transverse and longitudinal wave pulses in the micro-crack region (*c* = 0.009375, *f_R_* = 6 MHz, *μ* = 0, *L*_1_ = 40 mm, *L*_2_ = 4 mm, *L*_3_ = 36 mm). (**a**) Displacement contour in the *x* direction. (**b**) Displacement contour in the *y* direction.

The displacement contours of 2 MHz transverse wave pulses and 8 MHz longitudinal wave pulses propagating in the micro-crack region are shown in Figure 4 (U1 and U2 represent the displacements in *x* and *y* direction, respectively). It can be clearly seen that the length of the micro-crack is significantly smaller than the wavelength of the two primary waves. Meanwhile, when the two primary waves interact with micro-cracks, the waveforms of the two primary waves show no obvious change. The phenomenon of micro-cracks opening and closing can be verified when the deformation shapes are magnified 1500 times.

**Figure 5:**
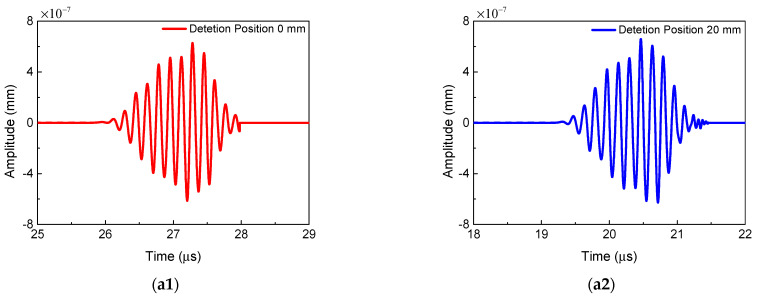

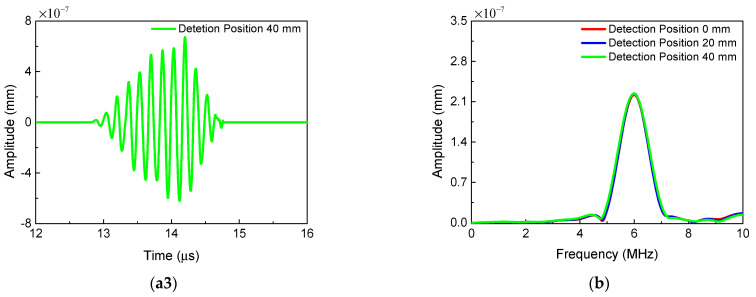
The signals of the resonant waves received at three signal detection positions (*c* = 0.009375, *f_R_* = 6 MHz, *μ* = 0, *L*_1_ = 40 mm, *L*_2_ = 4 mm, *L*_3_ = 36 mm). (**a1**–**a3**) Time-domain; (**b**) frequency-domain.

Figure 5 shows the resonant wave signals in the time-domain and frequency-domain received at three signal detection positions, which are 0 mm, 20 mm, 40 mm away from the left edge of the model, respectively. Because the size of the micro-crack region is smaller than the ideal duration of the resonant wave pulse, the received resonant wave signals present an incomplete diamond shape [16,20]. The frequency-domain signal of the resonant wave in Figure 5b indicates the notable existence of the 6 MHz resonant frequency. We can infer that the source of the nonlinearity leading to the generation of the resonant wave is the clapping and sliding behavior of micro-crack surfaces. Meanwhile, the time-domain and frequency-domain signals received at three signal detection positions are almost consistent. This proves that the signal of the resonant wave in propagation remains unchanged.

**Figure 6:**
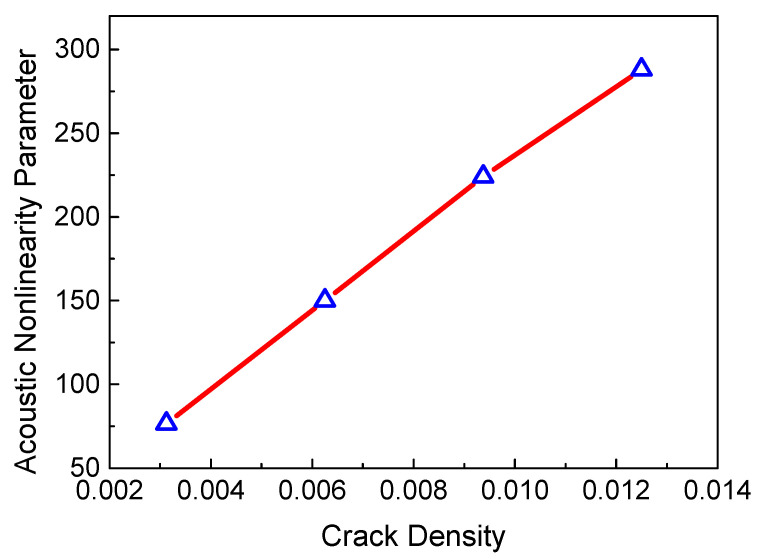
ANP versus crack density (*f_R_* = 6 MHz, *μ* = 0, *L*_1_ = 40 mm, *L*_2_ = 4 mm, *L*_3_ = 36 mm).

**Figure 7:**
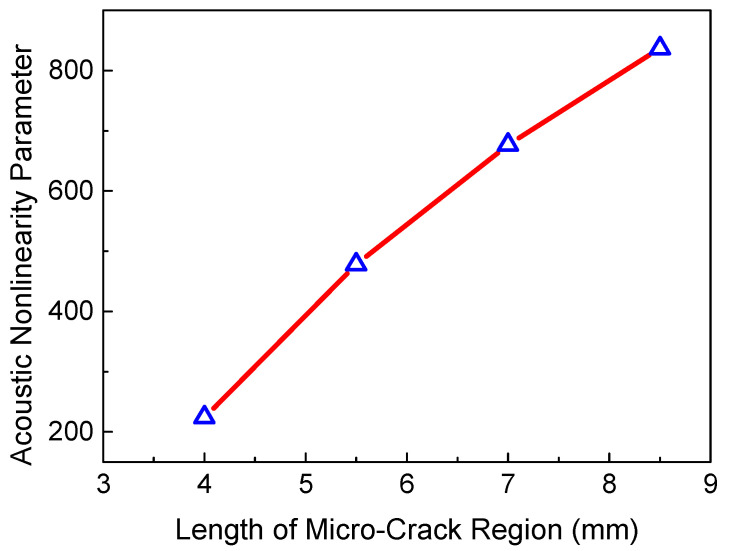
ANP versus the size of micro-cracks (*c* = 0.009375, *f_R_* = 6 MHz, *μ* = 0, *L*_3_ = 36 mm).

Next, four key factors were considered to investigate the relationship between the ANP and micro-cracks, such as the micro-crack density *c*, the size of the micro-crack region *L*_2_, the friction coefficient *μ* and the resonant frequency *f_R_*. Figure 6 shows the ANP versus the crack density. Based on the definition of crack density $c=Na^{2}/A$, the parameters *N* and *a* can both affect crack density. When the size of the micro-crack region, the resonant frequency and the friction coefficient remain the same, the ANP increases linearly with the crack density. A previous study [30] indicated that the effect of *N* and *a* on crack density is consistent. Considering the assumption of low frequency, parameter *a* needs to remain unchanged. Thus, we can increase the crack density by increasing the crack number *N*. The linear relationship between the ANP and the size of the micro-crack region is shown in Figure 7. It should be noted that the length of the micro-crack region is smaller than the ideal duration of the resonant wave pulses. The generated length of the resonant wave pulses can increase with the propagation and interaction of the two primary waves in the micro-crack region, which leads to the linear accumulation feature of the resonant wave.

**Figure 8:**
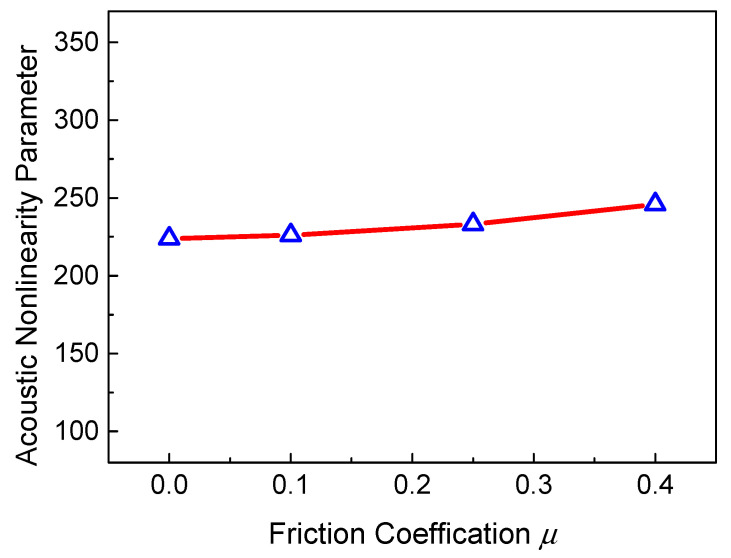
ANP versus friction coefficient (*c* = 0.009375, *f_R_* = 6 MHz, *L*_1_ = 40 mm, *L*_2_ = 4 mm, *L*_3_ = 36 mm).

Considering the interaction between micro-cracks and the two primary waves, not only the clapping behavior of micro-crack surfaces but also the slipping behavior may affect the generation of resonant waves. Figure 8 reveals the ANP versus the friction coefficient. It can be clearly seen that the relationship between the ANP and the friction coefficient has a slightly increasing trend. We can deduce that the clapping behavior plays the leading role in the contact interaction of micro-crack surfaces. Thus, the ANP is not sensitive to the friction coefficient.

**Figure 9:**
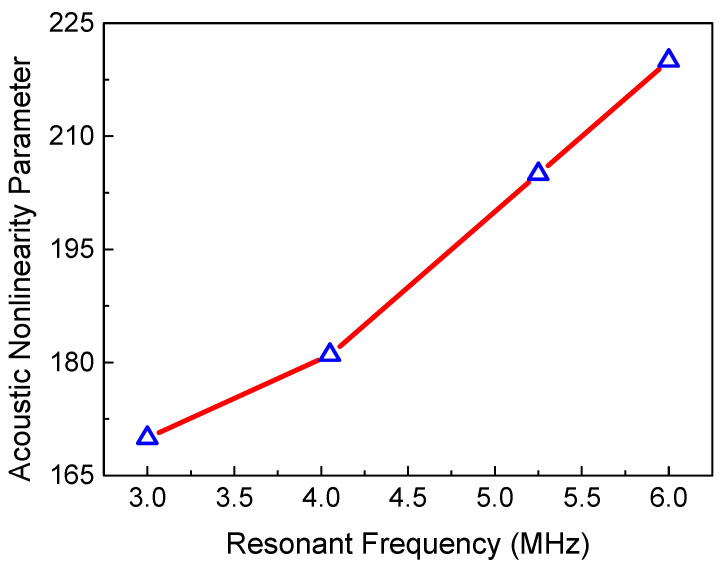
ANP versus the frequency of the resonant wave (*c* = 0.009375, *μ* = 0, *L*_1_ = 40 mm, *L*_2_ = 4 mm, *L*_3_ = 36 mm).

In addition, the ANP from micro-cracks could be affected by the frequency of the resonant wave. We found that the ANP represents a monotone function of the resonant frequency, as shown in Figure 9. Higher frequencies of two primary waves can lead to stronger interaction between these waves and micro-cracks, which can generate a resonant wave with a smaller wavelength and higher frequency. Then the resolution and sensitivity for detecting micro-cracks can be promoted with the increasing frequency of the resonant wave. Therefore, a resonant wave with higher frequency could provide better detection of micro-cracks.

**Figure 10:**
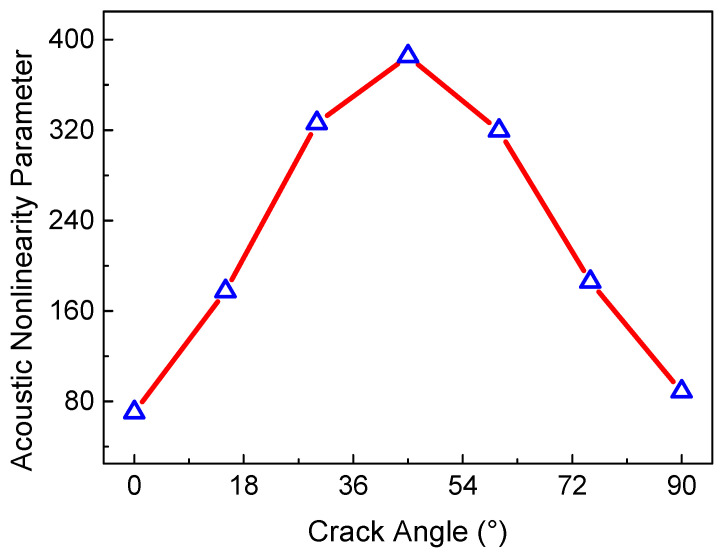
ANP versus crack angle (*c* = 0.009375, *f_R_* = 6 MHz, *μ* = 0.3, *L*_1_ = 40 mm, *L*_2_ = 4 mm, *L*_3_ = 36 mm).

**Figure 11:**
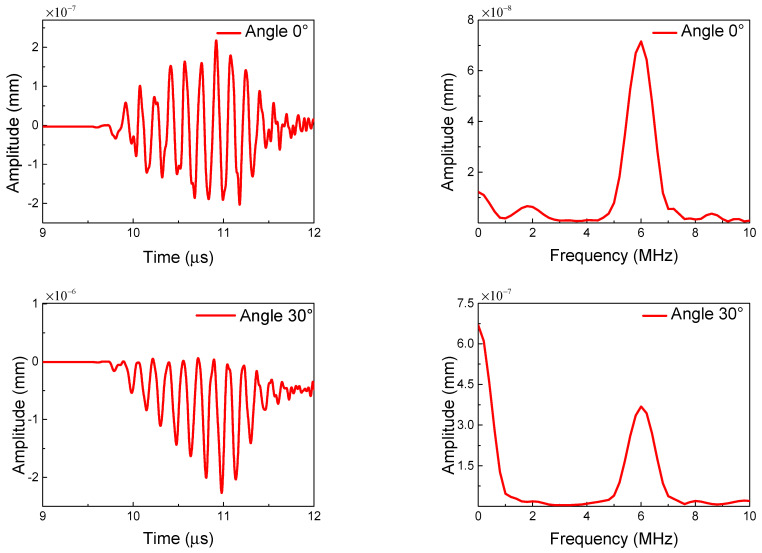

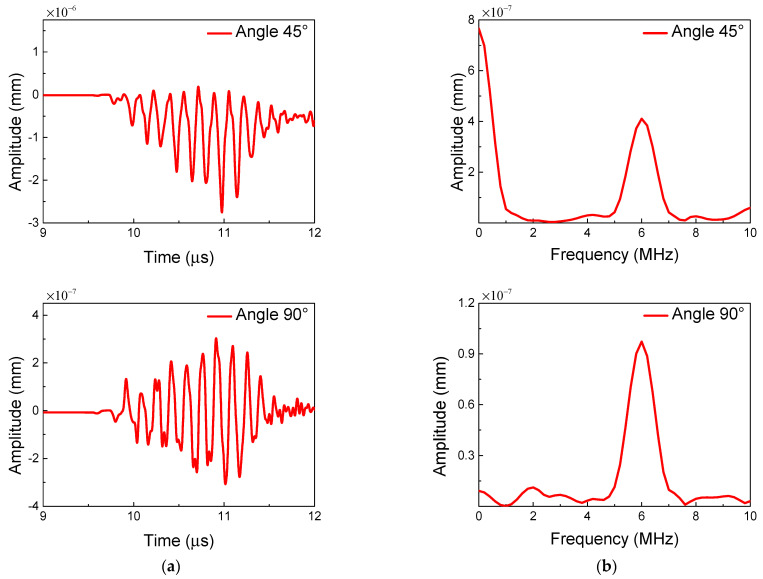
Waveforms (**a**) and frequency spectrums (**b**) of the resonant waves with different angles of micro-cracks (*c* = 0.009375, *f_R_* = 6 MHz, *μ* = 0.3, *L*_1_ = 40 mm, *L*_2_ = 4 mm, *L*_3_ = 36 mm).

Finally, when the orientation of all the micro-cracks keeps a certain angle, the mechanical property of the micro-crack region can possess anisotropy behavior. These cases need to be considered in engineering. Note that the position of the micro-cracks remains uniformly and randomly distributed. Figure 10 shows the relationship between the ANP and the micro-crack angle (the angle between the crack longitudinal direction and the *x* direction). It can be clearly seen that the trend in the ANP with the change in the micro-crack angle (in the range from 0° to 90°) is a bell-shaped curve. The maximum ANP is reached when the crack angle is about 45°, and the ANP decreases with the crack angle gradually deviating from 45°. The main reason for this phenomenon can be ascribed to the clapping behavior of micro-crack surfaces for both primary waves. When the micro-crack angle is close to 0°, the clapping behavior caused by the longitudinal wave tends to its minimum; conversely, when the micro-crack angle approaches 90°, the clapping behavior caused by the transverse wave tends to its minimum. Figure 11 shows the time-domain and frequency-domain in the representative crack angles. The waveform distortion is much more serious when the crack angle is close to 45°. The anisotropy in the micro-crack region caused by the uniform crack angle can lead to the invalidation of the resonant condition $\omega_{L}/\omega_{T}=2\kappa /\left(\kappa -1\right)$. Thus, the resonant wave is seriously affected by the material anisotropy caused by the uniformly aligned micro-cracks.

### 2.4. Method of Locating Micro-Crack Region

**Figure 12:**
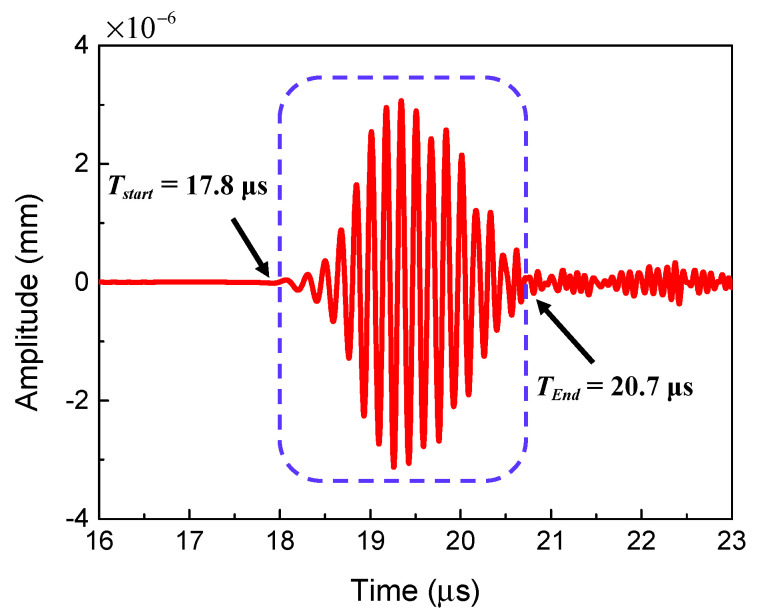
Time-domain signal of the resonant wave for locating the micro-crack region (*c* = 0.00625, *f_R_* = 6 MHz, *μ* = 0.3, *L*_1_ = 35 mm, *L*_2_ = 10 mm).

Compared to traditional ultrasonic nonlinear techniques, the wave mixing method has the important advantage of locating the micro-crack damage region. The location and the length of the micro-crack region were calculated by the time-domain signals of the two-way collinear mixing method as shown in Figure 12. The starting position *L*_1_ and the length *L*_2_ of the micro-crack region can be calculated by the following expressions:

(1) $$L_{1}=[(T_{Start}-T_{T}-\Delta T)C_{p}^{L}-L]C_{p}^{T_{R}}/\left(C_{p}^{L}-C_{p}^{T_{R}}\right)$$

(2) $$L_{2}=[(T_{End}-T_{T}-\Delta T-T_{L})C_{p}^{L}-L+L_{1}]C_{p}^{T_{R}}/C_{p}^{L}-L_{1}$$

where *T_Start_* and *T_End_* are the starting and ending time of the resonant wave, respectively, *T_T_* and *T_L_* are the time of exciting transverse wave pulse and longitudinal wave pulse, respectively, $C_{p}^{T_{R}}$ and $C_{p}^{L}$ are the velocities of the resonant and longitudinal primary waves, respectively, Δ*T* is the exciting time delay between the transverse wave and longitudinal wave, and *L* is the length of the model.

In addition, the location of the micro-crack damage region for different cases can be calculated and the results are shown in Table 1. It can be clearly seen that the simulation results agree well with the theoretical models. Therefore, the time-domain signal of the resonant wave can be employed to locate the micro-crack region.

## 3. Experimental Measurement

To investigate the validity of the two-way collinear wave mixing method for the detection of micro-cracks in practical engineering, a micro-crack damaged specimen was prepared through a low-temperature fatigue experiment. Note that the micro-crack damaged specimen is not strictly coincident with that used in the numerical simulations. Then the microstructure of the damage region was observed by a surface electron microscope (Model: Phenom XL, Phenom-World BV, Eindhoven, Netherlands). Meanwhile, ultrasonic measurement based on the two-way collinear wave mixing method was employed to detect the micro-crack damage by scanning the fatigue specimen.

### 3.1. Preparation of Micro-Crack Damage

**Figure 13:**
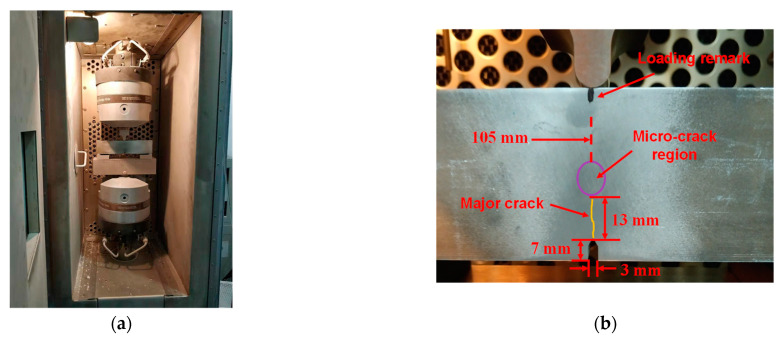
(**a**) The low-temperature three-point bending fatigue experiment. (**b**) The manufactured gap and the major fatigue-crack.

In the low-temperature fatigue test [58,59], the toughness of metals can be decreased while the brittleness can be increased, which can induce micro-cracks. Thus, a three-point bending fatigue experiment with a constant temperature of −40 °C was performed on the rectangle specimen (type of Al-5052) measuring 210 × 50 × 50 mm^3^, as shown in Figure 13. The fatigue testing machine was an MTS 809 axial/torsional test system (Model: MTS 809.10, MTS Systems Corporation, Eden Prairie, MN, USA) with a controlled environmental chamber. A notch (7 × 3 × 50 mm^3^) with a triangle tip was manufactured in the middle of the specimen to easily generate the major fatigue crack. Then the micro-crack damage region could be effectively induced in front of the major fatigue crack. The span of the bottom support in the three-point bending experiment was 200 mm and the top indenter was loaded on the middle of the upper surface of the specimen. Stress control (minimum pressure *F_min_* = −28 kN, stress amplitude *R* = 10 and loading frequency *f_load_* = 10 Hz) was employed in the three-point bending fatigue experiment. After 280,000 fatigue cycles, a major crack with a length of 13 mm was clearly visible.

**Figure 14:**
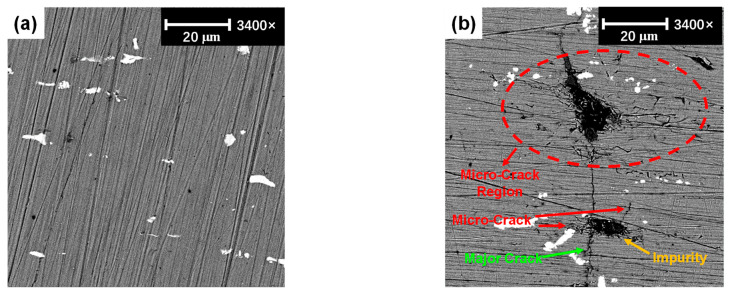
SEM images of the specimen. (**a**) The non-crack damage region. (**b**) The tip region of the major crack.

The microstructure of the major crack tip was observed by SEM, as shown in Figure 14, which shows the comparison between the non-damaged and damaged region at the same magnification of 3400. Note that to clearly observe the microstructure using SEM, the surface of the specimen was polished. Distinct and regular scratches can be found in the non-damaged and damaged regions. Besides, white spots can also be seen in Figure 14, which is typically caused by the production process of aluminum magnesium alloy [60]. Importantly, in the non-damaged region, no obvious damage with intact microstructures can be observed in Figure 14a. However, a distinct major crack can be observed in Figure 14b. The width of the major crack is rather small, which means it can be considered as a closed crack. Specifically, in the front of the major crack, it can be clearly seen that lots of micro-cracks exist around the tip of the major crack and impurities. The impurities could be deemed to be the source of micro-cracks. The representative size of the impurity is 20 μm, and the size of the micro-cracks ranges from 5 μm to 30 μm. Meanwhile, the major crack propagates across the impurity and the micro-cracks exist around the impurity in the middle bottom of Figure 14b, which indicates that the aggregation of micro-cracks could generate the macro-crack. This is in good agreement with the phenomenon described in [58].

### 3.2. Ultrasonic Measurement

**Figure 15:**
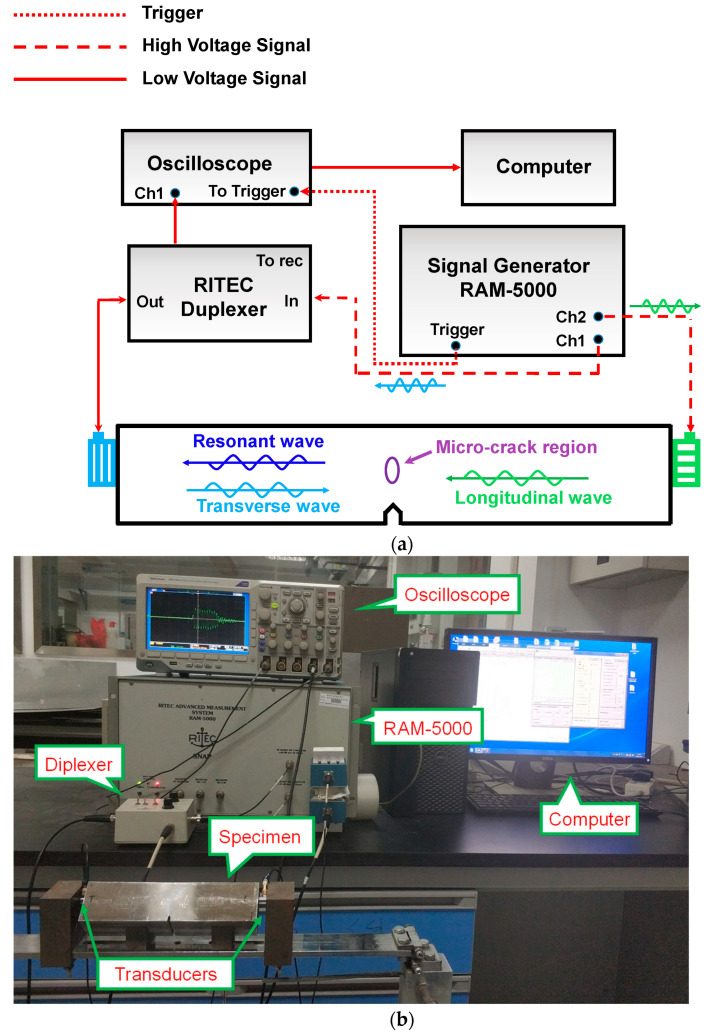
Experimental setup of the two-way collinear mixing method. (**a**)The schematic diagram. (**b**) Experimental installation.

Figure 15 shows the experimental setup of the two-way collinear mixing method for micro-crack damage detection. Two primary wave signals are generated by the high-power gated amplifier RAM-5000 SNAP (RITEC Inc, Warwick, RI, USA), and emitted by the transducers at two sides of the specimen. A resonant wave can be generated based on the two-way collinear mixing method and received by the transverse transducer. Then, the received signals could be saved as data by the DPO 3014 digital oscilloscope (Tektronix Inc., Beaverton, OR, USA). The reference trigger signal of the oscilloscope is from the internal trigger signal of RAM-5000 SNAP system.

In the experiment, the frequency pair of 2–8 MHz was chosen to satisfy the resonant condition. In order to launch the ideal sine wave signals, the transducers should be chosen carefully, especially for the transverse wave transducer, where the bandwidth should be enough wide to emit the primary transverse wave and receive the resonant wave. Thus, the 2 MHz transverse wave pulse with 10-cycles was triggered from channel 1 of the RAM-5000 SNAP system and excited by the left Olympus transverse transducer (Model: V155-RM, Olympus Inc., Tokyo, Japan). Similarly, the 8 MHz longitudinal wave pulse with 10-cycles was triggered from channel 2 and excited by the right longitudinal Olympus transducer (Model: V121-RM, Olympus Inc., Tokyo, Japan). It should be noted that the positions of the two transducers should be at the same height as the micro-crack region. In order to clearly observe the resonant wave, the output levels of two channels are both set as 100% to maximize the output energy. The two primary waves propagate in the specimen, mix and interact in the nonlinear region of the specimen. Then, a 6 MHz resonant transverse wave can be generated and received by the left transverse transducer. The RITEC duplexer (RITEC Inc, Warwick, RI, USA) was used to achieve the function of the transverse transducer, both transmitting and receiving. Finally, the received signals were digitized by the oscilloscope with a sampling frequency of 2.5 GHz and 100 k sampling points with 512 times average.

The resonant wave signal can be interfered with by electromagnetic signals and overlaid by the primary wave signals. Therefore, operations with the phase reverse method were executed to extract the resonant wave signals in the experiment. The received signal *S*1 was obtained by successively exciting the two primary waves with the positive phase, and the received signal *S*2 was obtained by successively exciting the two primary waves with the negative phase. Thus, the time-domain signal of the resonant wave can be acquired by the operation (*S*1 + *S*2)/2.

### 3.3. Experimental Results

**Figure 16:**
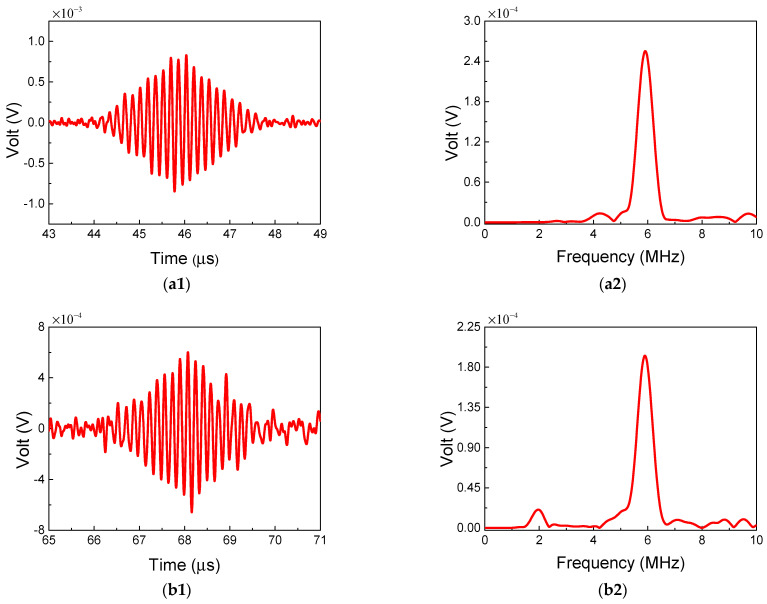
Time-domain and frequency-domain of experimental signals. (**a1**,**a2**) The resonant wave signal mixing at the position of 80 mm. (**b1**,**b2**) The resonant wave signal mixing at the position of 105 mm.

In this section, the validity of the two-way collinear mixing method for detection of the micro-crack region is discussed in detail. Figure 16 shows the representative time-domain and frequency-domain signals from two different mixing positions. Among them, Figure 16a1,b1 show the time-domain signals of the resonant wave mixing at the position of 80 mm and 105 mm away from the transverse transducer, respectively. When two primary waves mix at a position away from the micro-crack region, the resonant wave can be generated due to intrinsic material nonlinearity. Correspondingly, when two primary waves mix at a position within the micro-crack region, the resonant wave can also be generated due to quadratic nonlinearity and micro-cracks. Thus, the changes of the ANP could represent the local increase in the degree of damage. Meanwhile, the waveforms of the resonant waves show the typical diamond shapes, and the frequencies of the resonant waves are equal to the differences of the two primary waves. It can be clearly seen that the waveform of the resonant signal received at 105 mm is slightly distorted. The reason could be the reflected primary transverse wave caused by the macro-crack, which can be directly proved by the 2 MHz frequency component in Figure 16b2.

**Figure 17:**
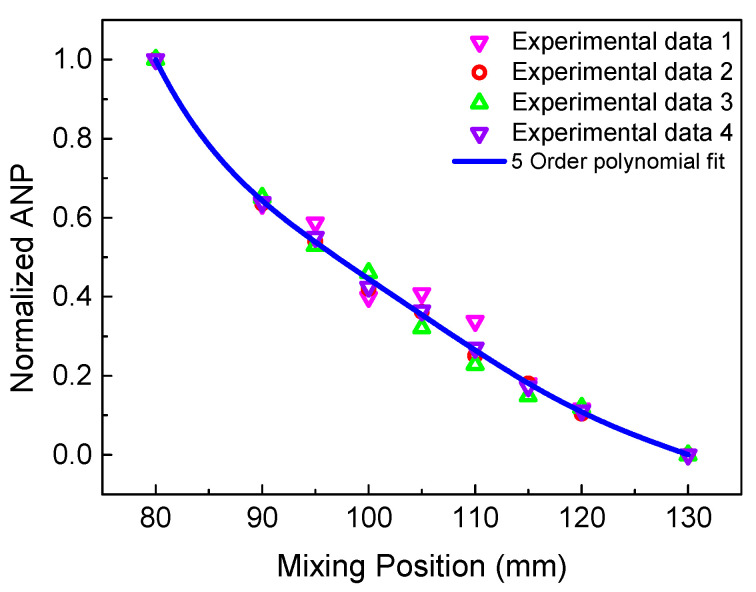
Normalized ANP of the 6 MHz resonant wave versus mixing position with non-fatigue experiment.

**Figure 18:**
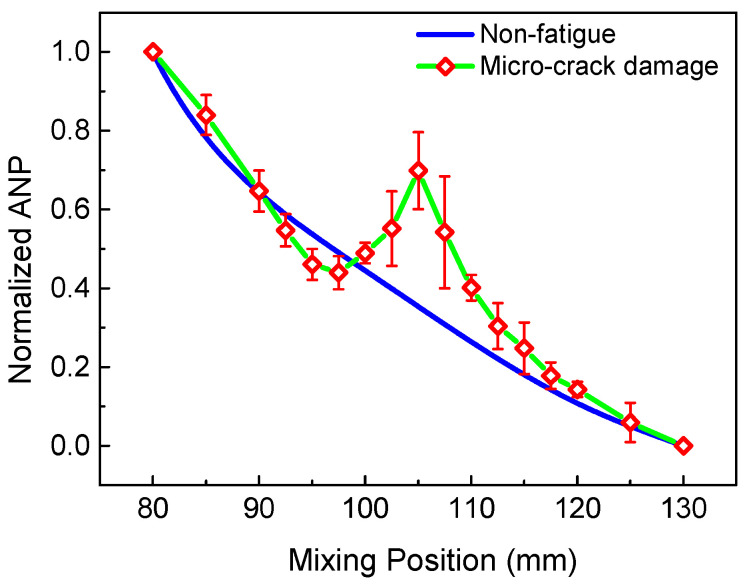
Normalized ANP of the 6 MHz resonant wave versus mixing position with non-fatigue and fatigue experiment.

**Figure 19:**
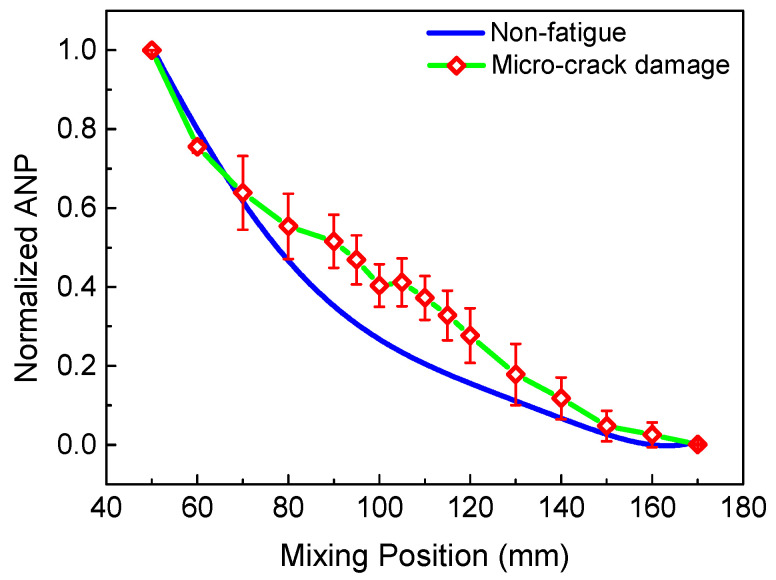
Normalized ANP of the 3 MHz resonant wave versus mixing position with non-fatigue and fatigue experiment.

Changing the time delay to emit the longitudinal wave, means the mixing region can be moved from the left to the right of the specimen. Then, the process of scanning the specimen can be achieved. Figure 17 reveals the normalized ANP of the 6 MHz resonant wave versus the mixing position. Notably, due to the beam spreading and the attenuation with the propagation distance, the received signals do not contain all resonant wave signals, which can result in the decrease in the resonant wave energy away from the mixing position. Thus, when the intrinsic material nonlinearity is uniformly distributed in the non-fatigue specimen, the relationship between the normalized ANP and mixing position could represent the trend in the signal intensity, which decreases smoothly [12]. Figure 18 shows a comparison of the scanning results for the fatigue specimen and the non-fatigue specimen, which agrees well with [21]. Compared with the non-damaged region, micro-crack damage can generate higher acoustic nonlinearity due to the clapping behavior. Thus, a significant increase of the normalized ANP can be observed in the middle region with the micro-crack damage. The position of the peak in Figure 18 is coincident with the location of the micro-crack damage in the fatigue experiment. Similarly, the scanning result using the 3 MHz resonant wave is shown in Figure 19. An inconspicuous increase of the normalized ANP is also obtained in the micro-crack damage region. The increment in the normalized ANP at the middle of the mixing position in Figure 19 is obviously smaller than that in Figure 18. Thus, we can conclude that the 6 MHz resonant wave has higher resolution and sensitivity than the 3 MHz resonant wave for locating the micro-crack damage region. The experimental phenomenon is consistent with the simulation conclusion regarding the resonant frequency in Figure 9. Therefore, the two-way collinear mixing method can effectively detect the micro-crack damage by scanning the specimen.

## 4. Conclusions and Discussion

In this study, a two-dimensional numerical model was built to investigate the resonant wave based on the two-way collinear mixing method in the resonant condition $\omega_{L}/\omega_{T}=2\kappa /\left(\kappa -1\right)$ and the validity of locating the micro-crack damage region was verified numerically and experimentally. The following conclusions can be drawn:

Firstly, micro-crack damage can be generated by a low-temperature fatigue experiment, and SEM results indicate the dominant role of micro-cracks in the microstructure of the damaged region. Ultrasonic scanning of the specimen based on the two-way collinear mixing method can locate the damaged region. Moreover, the higher-frequency resonant wave has higher resolution and sensitivity than the lower-frequency resonant wave for locating the micro-crack damage region. Thus, choosing a reasonable frequency for resonant waves is helpful to efficiently detect the micro-crack damage.

Next, if satisfying the resonant condition $\omega_{L}/\omega_{T}=2\kappa /\left(\kappa -1\right)$, resonant waves with the opposite propagation direction to the transverse waves can be generated when a pair of longitudinal and transverse waves interact in the micro-crack region by the two-way collinear mixing method. The numerical results also reveal that the ANP shows linear accumulation with the increasing of the micro-crack density and the size of the micro-crack region. The friction coefficient of micro-crack surfaces has a weak effect on the ANP and the frequency of the resonant wave is associated with the ANP in a form of monotonous increase. Therefore, the ANP is feasible for effectively characterizing the micro-crack damage. More importantly, the location of the micro-crack damage region can be calculated by the start and end time of the resonant wave signals in the time-domain.

In summary, this paper experimentally verified the feasibly of the two-way collinear mixing method for the detection of micro-crack damage, and numerically investigated the characteristics between resonant waves and the micro-crack damage. Thus, this study provides the theoretical and experimental foundation for developing a nondestructive evaluation technique for micro-crack damage, especially for key engineering structures (such as aircraft, high-speed trains) under low-temperature fatigue loading. In further work, we will investigate more factors for the two-way collinear mixing method, such as the non-uniform distribution of micro-cracks in numerical simulations, and different temperature and fatigue life in fatigue experiments.

## Figures and Tables

**Table 1:** The location results of different cases.

	Simulation Results		Theoretical Models		Errors	
*L* (mm)	*L*_1_ (mm)	*L*_2_ (mm)	*L*_1_ (mm)	*L*_2_ (mm)	*L*_1_ (%)	*L*_2_ (%)
50	21.24	5.05	21	5	1.14	1.20
50	26.08	4.12	26	4	3.10	3.00
80	34.98	9.83	35.00	10	0.05	1.70
